# Visual EMDR stimulation mitigates acute varied stress effects on morphology of hippocampal neurons in male Wistar rats

**DOI:** 10.3389/fpsyt.2024.1396550

**Published:** 2024-05-13

**Authors:** Yaveth Ruvalcaba-Delgadillo, Diana Emilia Martínez-Fernández, Sonia Luquin, Ana Moreno-Alcázar, Diego Redolar-Ripoll, Fernando Jauregui-Huerta, David Fernández-Quezada

**Affiliations:** ^1^ Neuroscience Department, University Center of Health Sciences, University of Guadalajara, Guadalajara, Jalisco, Mexico; ^2^ Transdisciplinary Institute of Research and Services (ITRANS), University of Guadalajara, Zapopan, Mexico; ^3^ ISOMAE Institute of Neurosciences and Psychosomatic Psychology, Sant Cugat del Vallés, Spain. Centre Fòrum Research Unit, Hospital del Mar, Barcelona, Spain; ^4^ Cognitive NeuroLab, Universitat Oberta de Catalunya (UOC), Barcelona, Spain; ^5^ Laboratorio de Fisiología del Comportamiento, Departamento de Fisiología, Facultad de medicina, Universidad Nacional Autónoma de México, Ciudad de México, Mexico

**Keywords:** stress, EMDR, hippocampus, Golgi-Cox, Sholl analysis

## Abstract

**Introduction:**

Stress is a pervasive health concern known to induce physiological changes, particularly impacting the vulnerable hippocampus and the morphological integrity of its main residing cells, the hippocampal neurons. Eye Movement Desensitization and Reprocessing (EMDR), initially developed to alleviate emotional distress, has emerged as a potential therapeutic/preventive intervention for other stress-related disorders. This study aimed to investigate the impact of Acute Variable Stress (AVS) on hippocampal neurons and the potential protective effects of EMDR.

**Methods:**

Rats were exposed to diverse stressors for 7 days, followed by dendritic morphology assessment of hippocampal neurons using Golgi-Cox staining.

**Results:**

AVS resulted in significant dendritic atrophy, evidenced by reduced dendritic branches and length. In contrast, rats receiving EMDR treatment alongside stress exposure exhibited preserved dendritic morphology comparable to controls, suggesting EMDR’s protective role against stressinduced dendritic remodeling.

**Conclusions:**

These findings highlight the potential of EMDR as a neuroprotective intervention in mitigating stress-related hippocampal alterations.

## 1 Introduction

Stress represents a significant health challenge, triggering physiological responses that encompass various bodily systems, including metabolism, immune response, and reproduction (1–4). The brain exhibits remarkable adaptive capabilities through neuroplastic changes, including modifications in dendritic and synaptic morphology, allowing for dynamic responses to internal and external stimuli (5). Notably, intense stress can induce alterations in dendritic branching and synaptic connectivity, particularly affecting brain regions like the hippocampus (6, 7).

The hippocampus, vital for memory and stress response regulation, is particularly vulnerable to the effects of stress (6, 7). Acute stressors have been shown to elicit a cascade of neurobiological changes in the hippocampus, including alterations in neurotransmitter levels, disruption of neurogenesis, and impairment of synaptic plasticity (8, 9). Chronic or severe acute stress can lead to neuronal damage and atrophy within the hippocampal region, specifically in the CA3 and dentate gyrus subfields, as evidenced by reduced dendritic arborization and decreased spine density (10). These structural changes are often accompanied by functional deficits, such as impaired spatial memory and decreased resilience to further stressors (8, 11, 12).

Eye movement desensitization and reprocessing (EMDR) is a psychotherapeutic approach originally developed to eliminate emotional distress resulting from traumatic memories (13). This therapeutic technique was first used by Francine Shapiro in 1980’s and combines imagined exposure and other techniques to reduce the intensity of distressing thoughts and feelings (14). EMDR positive effects have been mainly attributed to bilateral sensory stimulation typically elicited by the therapist`s hand movements that induce lateral eye movements while the patient recalls distressing memories. Bilateral stimulation may facilitate information processing and adaptive memory consolidation, thereby reducing emotional distress and promoting psychological resilience (15, 16). Experimental research, including meta-analyses, systematic reviews, and clinical trials, consistently supports the efficacy of Eye Movement Desensitization and Reprocessing (EMDR) as a treatment for post-traumatic stress disorder (PTSD) and related conditions (17, 18). Although EMDR has proven to be effective, the intricacies of its mechanisms remain unknown, in part due to the lack of animal studies. Animal models are indispensable in medical research as they offer insights into physiological and pathological processes, mirroring those in humans. Animal models, particularly rats, are indispensable in medical research because they share many physiological and pathological traits with humans, as extensively documented in toxicological and other biomedical studies. These models offer invaluable insights into human biological processes and disease mechanisms because they replicate human organs and tissues (19). The use of rats is particularly prevalent in studies involving complex physiological responses, such as those observed in burn treatment research, due to their cost-effectiveness and the breadth of available histopathological data (20). This is particularly critical for EMDR, where understanding the neurological underpinnings could significantly enhance therapeutic outcomes.

This study aims to provide neuroanatomical evidence of EMDR´s effects using an Acute Variable Stress (AVS) rat model. We hypothesized that EMDR would reduce the effects of stress exposure on the dendritic tree of hippocampal neurons.

## 2 Materials and methods

### 2.1 Animals

**Figure 1:**
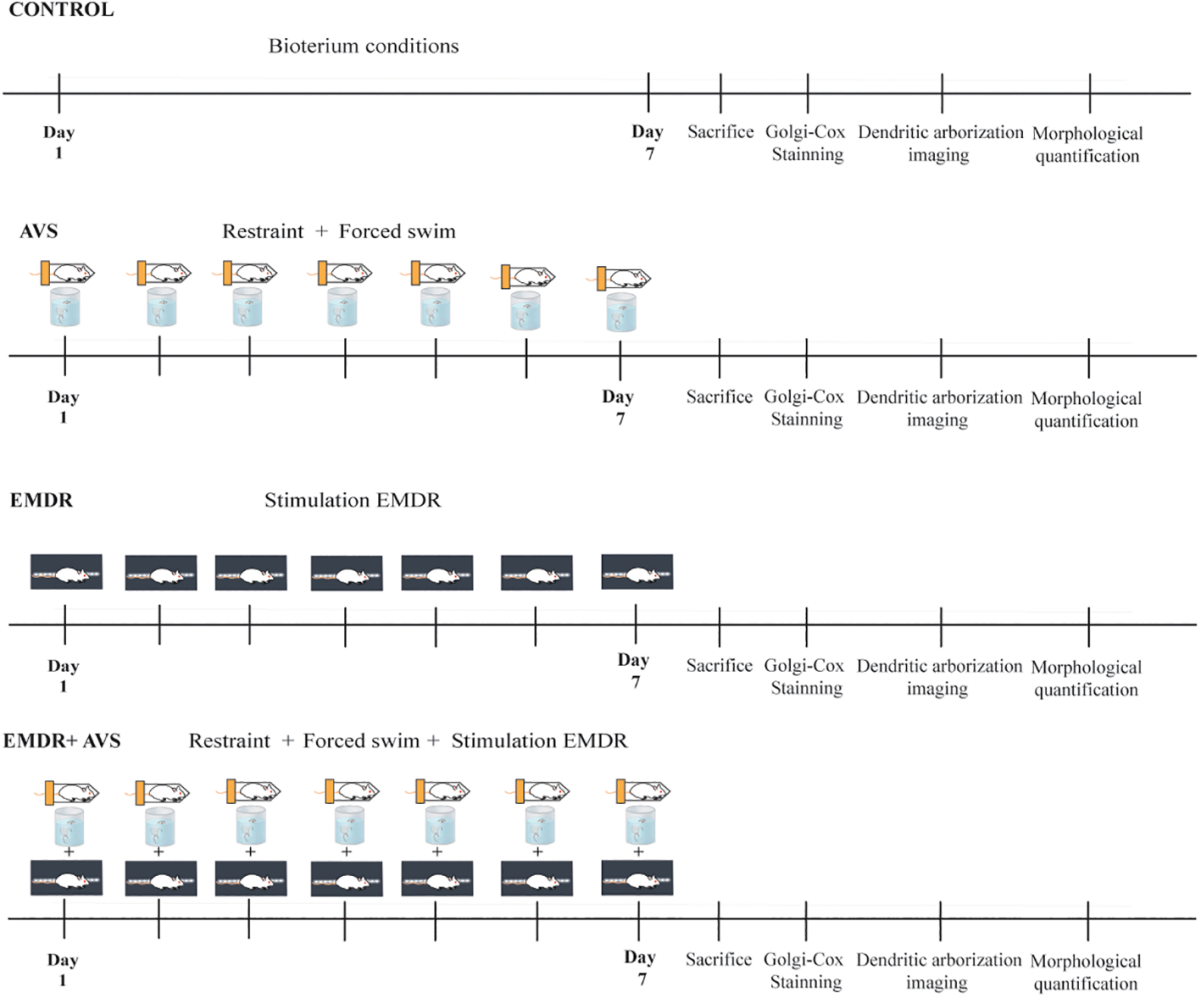
This shows the main procedures of the experiments carried out. Control group (CONTROL), Variable Stress (AVS), Eye Movement Desensitization and Reprocessing group + Acute Variable Stress (EMDR + AVS), and an Eye Movement Desensitization and Reprocessing group (EMDR).

In this study, 90-day-old Wistar male rats were employed. A total of 20 male rats, weighing 299.4 ± 26.41g, were randomly allocated to groups using a computer-generated sequence (Research Randomizer, https://www.randomizer.org) to ensure equal distribution among groups, with the process blinded to minimize bias into four groups: a Control group (n = 5), EMDR group (n = 5), AVS (n = 5), and an EMDR + AVS (n = 5). All groups were maintained in a 12:12 light–dark cycle, with lights on at 7:00 am. The temperature in the experimental room was maintained at 25 ± 2°C, and humidity at 70%. The animals had free access to tap water and balanced food. The cages were changed in the testing room every day at 13:00h. All animal experiments followed the National Institute of Health guide for the care and use of laboratory animals, and the study was approved by the Health Sciences Ethics, Biosafety, and Scientific Board, at the University of Guadalajara, México CI. 068-2014. **Figure 1** illustrates all experimental procedures.

### 2.2 Acute variable stress protocol

The stimulus involved a sequential exposure to two distinct stressors over a seven-day period, starting with spatial restriction. Each rat was confined in an 8x18 cm PET plastic cylinder, equipped with 5 mm ventilation holes, for 30 minutes to limit movement while ensuring adequate ventilation, aiming to induce physical stress. Following this, to introduce psychological stress, rats underwent a forced swim in a 45 cm high, 30 cm diameter plexiglass cylinder filled with water at 10°C for 15 minutes, with a 10-minute rest interval between the stressors. This regimen was conducted daily at 9:00 am to minimize circadian variations, in contrast to a control group was remained unexposed to stressors and was kept under standard laboratory conditions. The term ‘variable stress’ was used to represent the combination of physical and psychological stressors, rather than a variation in the stressors. The term exposure as ‘acute’ was established on our interpretation in contrast with other protocols that employed a 21-day model for chronic stress (21–23). Despite potential ambiguities in distinguishing between acute, sub-acute, and short-term chronic stress, this classification is validated by our prior findings (24).

### 2.3 Eye movement desensitization and reprocessing stimulation

The EMDR device used in the study facilitated simultaneous stimulation of up to five rats. It had dimensions of 40 x 75 x 30 cm, with individual compartments for rats measuring 40 x 15 x 30 cm each. These dimensions were selected to allow for a certain level of space restriction, enabling rats to observe LEDs installed in the box from any position without complete immobilization. During experiments, rats were closely monitored to ensure that received light stimulus.

Upon activation, the EMDR device emitted light stimuli through 20 intermittently flashing LEDs at a frequency of 1Hz (25). These LEDs sequentially turned on and off from one end of the device to the other and vice versa controlled by an electrical circuit consisting of an NE555 timer, 3 resistors of 1 kΩ, and a 100 μF capacitor at 16 V, regulated by a 10 kΩ potentiometer. The LED strip was positioned 10 cm from each end of the box to ensure visibility from any position.

After completing each day of acute variable stress protocol, rats from the EMDR and EMDR + AVS groups were placed in the EMDR box in darkness, away from distractions. We used the same room and conditions than previously used to induce stress in order to elicit distressing memories. They were given 5 minutes to familiarize themselves with the EMDR box for environmental recognition. Subsequently, the stimulus was applied through two 1-minute sessions, with a 30-minute rest period between sessions.

### 2.4 Golgi-Cox staining

**Figure 2:**
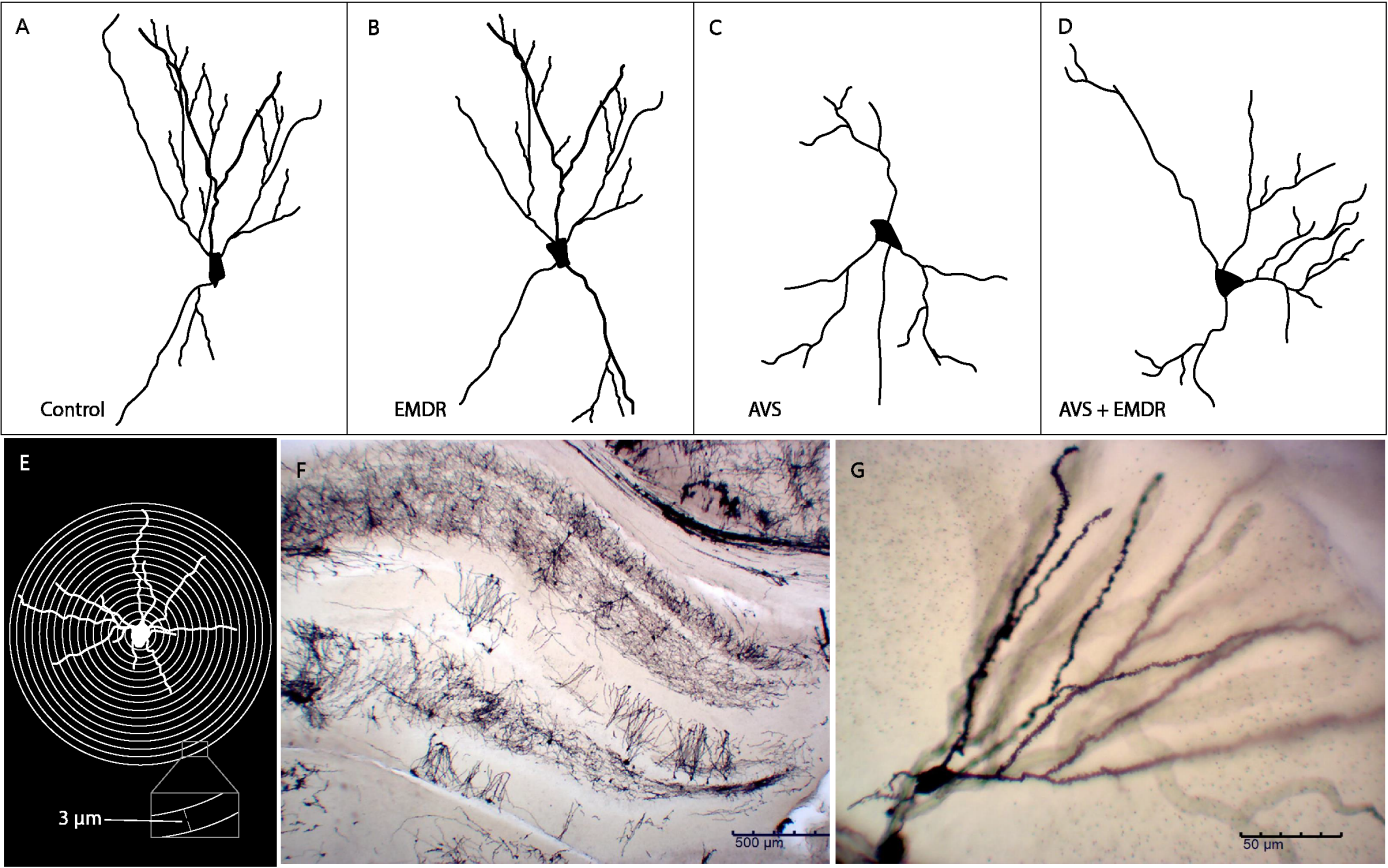
Schematic representation of neuronal dendritic morphology. **(A)** Control group; **(B)** Eye Movement Desensitization and Reprocessing group (EMDR); **(C)** Acute Variable Stress group (AVS); **(D)** Acute Variable Stress group + Eye Movement Desensitization and Reprocessing group (AVS +EMDR); **(E)** Sholl analysis; **(F)** Panoramic microscopy photograph of the hippocampus stained with Golgi-cox; **(G)** Representative microscopic photograph 20X (1296×972, 0.22 px/μm) of Golgi-cox staining.

Immediately after the animals completed the experimental procedure, they were anesthetized and decapitated. The skulls were opened, and the brains were quickly removed, cut with a blade into 1 cm thick slabs, and processed using a FD Rapid GolgiStain™ kit (FD Neuro Technologies, Ellicott City, MD, USA PK401 CAT# PK401). In each procedure, blocks from all rat groups were put in the dark at room temperature into a mixture of solutions (provided by the kit producer) for the next 2 weeks. The tissues were then transferred into a protectant solution C (0.1 M phosphate buffer, sucrose, polyvinylpyrrolidone and ethylene glycol) and stored for 48 hours in the dark at 4°C. The tissues were sectioned into 120 μm slices using a vibratome (Leica, VT1000 S). Each section was mounted with a protectant solution on gelatin coated microscope slides. Sections were then dried at room temperature in the dark for a couple of hours. For the next procedure the slides were collocated into the staining solution D and E (ammonia and sodium thiosulfate). Then the tissues were dehydrated in 50%, 75%, 95% and 100% ethanol and cleared in xylene. The tissues were coverslipped in Permount™ Mounting Medium. The slides were finally viewed under a Leica DMi8 microscope (Leica Microsystems Inc, Bannockburn, IL), **Figures 2F, G**.

### 2.5 Dendritic arborization imaging

The neuronal tree arborization imaging was performed on hippocampus sections encompassed between Bregma 3.72 mm and Bregma −4.68 mm. Positive Golgi-Cox staining was localized and photographed in all hippocampal cortex regions. Briefly, distal apical and basal dendrites of neurons were imaged using a Leica DMi8 microscope (Leica Microsystems Inc, Bannockburn, IL) with a 20× objective lens. Images were acquired with a DFC 7000T Leica camera, at 1296×972 pixels in the x- and y-axis (0.22 px/μm). Inclusion criteria for the dendritic quantification involved neurons that were fully intact, with clearly distinguishable dendritic structures observable within the designated hippocampal region. Neurons were also required to exhibit no signs of damage or pathological abnormalities. Exclusion criteria encompassed neurons with incomplete dendritic trees due to slicing or processing artifacts, as well as those showing signs of degeneration or any form of morphological distortion that could interfere with accurate measurement. This stringent selection process was implemented to ensure that only neurons suitable for accurate and representative analysis were included in the study.

### 2.6 Morphological quantification analysis

The neurons were processed according to the Sholl methodology (23, 26, 27). It consisted of image acquisition, skeletonization, generation of meta-data, quantification, analysis, and interpretation. The semi-automated tools available through the NeuronJ plugin (28) to ImageJ (NIH, Bethesda, MD) were used to define the positions of all neurites. Then, Matlab scripts were employed to convert the strings of nodes provided by NeuronJ into SWC format NeuronStudio (29) and then used to define the pattern of connectivity between neurite segments. Finally, the Bonfire scripts of Matlab´s software were applied to integrate neuronal digitization (NeuronJ and NeuronStudio) and extract Sholl profiles. We analyzed 50 neurons per group and quantified the number of intersections in concentric rings at 3 μm intervals (Sholl analysis). **Figure 2E** illustrate the total number of dendrites and the total length of dendrites. **Figures 2A-D** shows a representative digitalized neuron from each group.

### 2.7 Statistical analysis

Data measurements were averaged and the significance of the differences between groups of rats were tested by one-way ANOVA. All statistical analysis was performed using GraphPad (GraphPad, version Prism 8). Data were expressed as mean ± SEM. *Post hoc* test analysis (Holm–Sídák analysis to correct multiple comparisons) was employed to explore differences in single time points between groups. Differences were considered statistically significant at a value * p < 0.05 (** p < 0.01, *** p < 0.001).

## 3 Results

### 3.1 Effects of AVS and EMDR stimulation on hippocampal dendritic complexity

**Figure 3:**
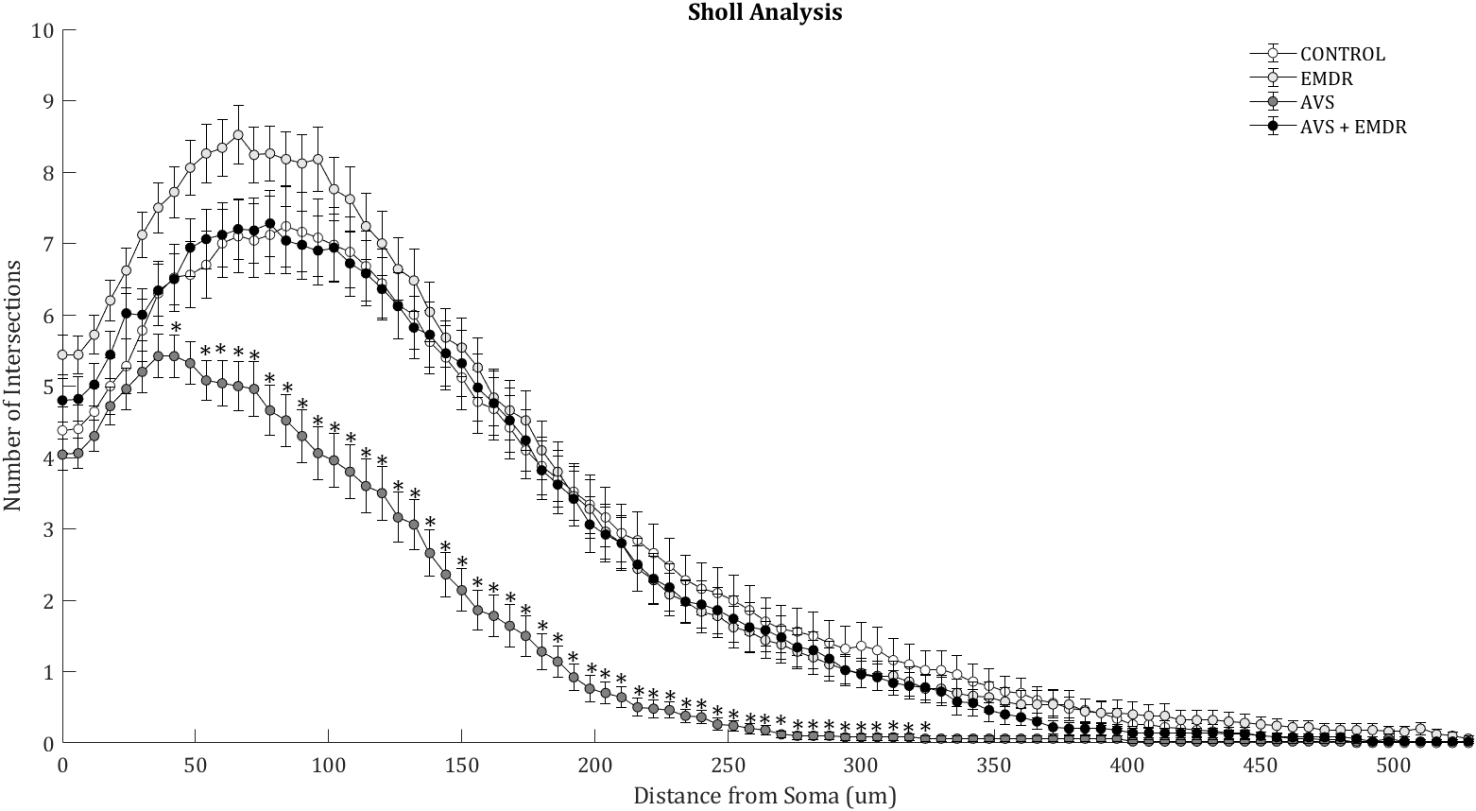
Morphometry of hippocampal neurons. Illustrates the Sholl analysis with the number of intersections found in 50 neurons per group. Control group; Eye Movement Desensitization and Reprocessing group (EMDR); Acute Variable Stress group (AVS); Acute Variable Stress group + Eye Movement Desensitization and Reprocessing group (AVS +EMDR). Data represent the mean ± SEM. *p < 0.05 (**p < 0.01, ***p < 0.001).

ANOVA analysis revealed significant differences in Sholl analysis [F(3, 196) = 20.55; p < 0.0001]. The number of intersections in the AVS group decreased significantly (126.7 ± 9.01 & 242.3 ± 16.66; p < 0.001), in comparison to the control group. Also, in the AVS +EMDR group a statistically significant increase was exhibited (126.7 ± 9.01 & 236.3 ± 13.91; p < 0.001), in contrast to the AVS group, **Figure 3**.

**Figure 4:**
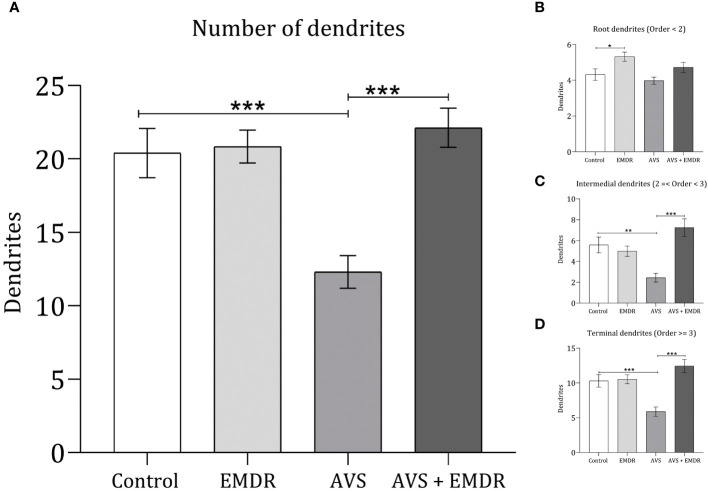
Dendritic complexity in hippocampal neurons. Control group; Eye Movement Desensitization and Reprocessing group (EMDR); Acute Variable Stress group (AVS); Acute Variable Stress group + Eye Movement Desensitization and Reprocessing group (AVS +EMDR). Data represent the mean ± SEM. *p < 0.05 (**p < 0.01, ***p < 0.001).

The quantification of dendritic arborizations in AVS group revealed a substantial reduction (12.30 ± 1.11 & 20.40 ± 1.67; p < 0.001) **Figure 4A**, specifically evident in the intermediate (2.44 ± 0.41 & 5.58 ± 0.75; p < 0.0024) **Figure 4C**, and terminal dendritic segments (5.88 ± 0.66 & 10.30 ± 0.90; p < 0.0004) **Figure 4D**, as compared to the control group [F(3, 196) = 11.27; p < 0.0001]. Notably, in the AVS + EMDR experimental group, there were no statistically significant differences in dendritic counts when contrasted with the control group (22.12 ± 1.332 & 20.40 ± 1.676; p = 0.5931). However, when analyzed against the AVS group, a marked increase in dendritic count was observed in the AVS + EMDR group (2.44 ± 0.41 & 7.24 ± 0.85; p < 0.001), particularly within the intermediate (12.30 ± 1.11 & 22.12 ± 1.33; p < 0.001), and terminal dendritic subpopulations (5.88 ± 0.66 & 12.44 ± 0.94; p < 0.001) **Figures 2**, **4**.

**Figure 5:**
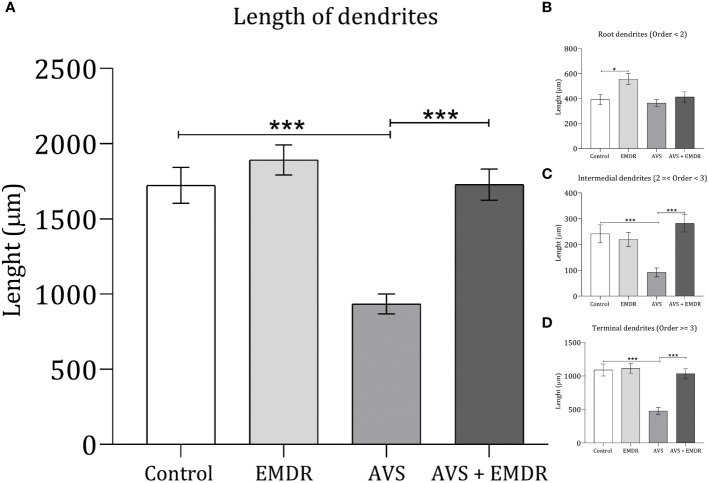
Length dendritic complexity in hippocampal neurons. Control group; Eye Movement Desensitization and Reprocessing group (EMDR); Acute Variable Stress group (AVS); Acute Variable Stress group + Eye Movement Desensitization and Reprocessing group (AVS +EMDR). Data represent the mean ± SEM. *p < 0.05 (**p < 0.01, ***p < 0.001).

In line with the dendritic count results, when assessing the overall length of the dendrites [F(3, 196) = 18.86; p < 0.0001], it was observed that the AVS group demonstrated shorter lengths (934.4 ± 66.04 & 1723 ± 119.1; p < 0.001) **Figure 5A**, both in the intermediate (91.70 ± 17.12 & 241.7 ± 35.11; p < 0.0010) **Figure 5C**, and terminal segments (478.8 ± 53.94 & 1090 ± 89.77; p < 0.001) **Figure 5D**. Similarly, the AVS + EMDR group exhibited significantly longer dendritic segments (934.4 ± 66.04 & 1727 ± 103.5; p < 0.001) at both the intermediate (91.70 ± 17.12 & 282.2 ± 33.40; p < 0.001), and terminal levels (478.8 ± 53.94 & 1033 ± 74.68; p < 0.001), compared to the AVS group, **Figures 2**, **5**.

## 4 Discussion

In this experiment, we investigated the impact of visual stimulation through EMDR on dendritic morphology in the hippocampal neurons of male rats subjected to acute variable stress.

First, our data evidenced that exposure to AVS led to significant dendritic atrophy in hippocampal neurons, evidenced by reductions in both the number of secondary and tertiary branches and their dendritic length. We believe that, since the stimulus was acute, changes were limited to intermediate and terminal dendrites, which could be reversible. This contrasts with studies of chronic stress over 21 days, where significant damage is reported in the apical neuronal dendritic morphology of hippocampus, with permanent alterations in its structure (30). These findings corroborate previous research demonstrating the detrimental effects of chronic stress on hippocampal structure and function (31).

The hippocampal formation has consistently been reported as highly susceptible to damage from various stressors, which was a primary reason for its inclusion in our experiment. This susceptibility arises primarily due to the intricate interplay between stress-related signaling pathways, notably the hypothalamic-pituitary-adrenal (HPA) axis (32–34). Consequently, the impact of stress on the hippocampus is regulated by several mediators, influencing brain function across different spatial and temporal scales. Glucocorticoids, for instance, are released in response to stress and can have widespread effects on brain function, often leading to elevated levels of these hormones (35).

Numerous studies have provided evidence supporting the detrimental effects of stress on hippocampal structure and function (36). For instance, chronic stress exposure has been shown to lead to significant dendritic retraction and synaptic loss in the CA3 region of the hippocampus in rodents (37). Similarly, other experiments found that chronic stress induced hippocampal atrophy and impaired spatial memory performance (33, 38). Glucocorticoids have the potential to impact plastic remodeling, particularly when stress persists over extended periods (30, 39). Specific experimental conditions have been demonstrated to induce CA3 dendritic retraction, including 21 days of predator stress (40), 6 days of activity stress (41), 28 days of unpredictable stress (42, 43), 14 days of social defeat stress (44, 45), 10 days of restraint stress (35), and 21 days of acoustic stress (27). The detrimental effects of acute variable stress (AVS) described in our experiment are thus well supported.

The hippocampus was chosen for our analysis not only due to its susceptibility to stress-induced damage but also because of its crucial role in learning and memory processes (46–48). It is well-established that the hippocampus is closely implicated in the pathophysiology of trauma and stress-related disorders, such as post-traumatic stress disorder (PTSD) and major depressive disorder (MDD). Individuals with PTSD often exhibit hippocampal volume reductions and alterations in hippocampal function, particularly in relation to memory encoding and retrieval processes (49, 50). Similarly, structural and functional abnormalities in the hippocampus have been observed in individuals with MDD. There is evidence that stress via the hypothalamic-pituitary-adrenal axis can result in elevated glucocorticoid levels and binding with glucocorticoid receptors in the hippocampus. As a result, neuronal atrophy occurs (51, 52). These effects have been reported as a smaller hippocampus associated with decreased brain activity, resulting in reduced gray matter volume, and reduced functional activity leading to negative emotions and impaired cognitive processing (53–55). These suggest a bidirectional relationship between hippocampal dysfunction and depressive symptoms (56). Therefore, our model closely mimics conditions where stress and cognition, such as learning and memory, interact to shape hippocampal structure and validate it to explore preventive treatments. As exposed before, EMDR represents a promising intervention for trauma and distressing memories.

In this context, we found that the EMDR-treated group exposed to the same stress condition did not exhibit dendritic retraction, maintaining identical levels of dendritic morphology as the control group. Furthermore, the group of rats receiving only EMDR stimulation showed an increase in both the number of primary dendritic branches and their length. Therefore, visual EMDR stimulation appears to mitigate the effects of stress on the dendritic architecture of hippocampal neurons.

EMDR stimulation has emerged as a therapeutic intervention specifically designed to address traumatic memories and associated stress symptoms, demonstrating efficacy in facilitating the processing of traumatic events (14, 57, 58). Despite its clinical success, the precise neurobiological mechanisms underlying trauma reprocessing through EMDR stimulation remain elusive. One prevailing hypothesis posits that bilateral stimulation may induce alterations in cognitive processing centers within the brain, facilitating the establishment of connections between past adverse experiences and responses to current non-traumatic stimuli (59). This bilateral stimulation is believed to elicit a relaxation response and trigger physiological reactions that, when integrated with stored information about prior adverse experiences, generate novel information in a functional manner (59–61). Despite various proposed theories, a unified understanding of EMDR therapy mechanisms remains lacking due to insufficient evidence.

Recently, Baek and colleagues conducted a groundbreaking study utilizing a mouse model to investigate the neurobiological mechanisms underlying EMDR therapy for the first time. In their research, animals conditioned to fear responses through sound paired with unpleasant electrical shocks exhibited reduced fear of the traumatic context following EMDR therapy. This reduction in fear was accompanied by a decrease in neuronal excitability within circuits involving the basolateral amygdala (62), providing pioneering evidence of the neurobiological effects of EMDR in a murine model. Furthermore, Mattera and colleagues propose a neural network model in PTSD and EMDR therapy that involves four areas representing the role of sensory cortices, the hippocampus, the amygdala, and the PFC (63). Moreover, clinical studies in humans have demonstrated the effectiveness of EMDR therapy in treating various populations, including infants with post-traumatic stress symptoms (64, 65), adults with post-traumatic stress disorder (66, 67), and patients resistant to depression treatment (68). A recent study reporting decreased levels of salivary cortisol in EMDR responder PTSD patients suggests that EMDR therapy may positively regulate cortisol levels and for this mechanism mitigate the impact of stress on hippocampal neurons (69). Also, it has been reported that the rise in limbic activity, characterized by heightened activation of the amygdala during the handling of negative emotional stimuli, coincides with a noted decline in activation within dorsolateral prefrontal brain regions associated with cognitive control mechanisms (70). However, additional experiments are warranted to investigate this hypothesis.

Furthermore, our findings represent the first histological experiment reporting the effects of EMDR therapy in a rat model. Our novel findings provide promising insights into conditions related to stress and trauma in humans. Demonstrating the impact of EMDR on neuronal structures within a controlled animal model allows us to propose potential neurobiological pathways through which EMDR could have therapeutic effects in humans. This discovery opens new avenues for future research aimed at exploring the neurobiological mechanisms of EMDR therapy. Nonetheless, must be approached with caution, acknowledging the gap between animal models and human clinical realities. These limitations highlight the need for further research to bridge these gaps and enhance the translational potential of our findings.

Future research will aim to incorporate hormonal and behavioral measures, offering a more comprehensive view of the stress response and the therapeutic potential of EMDR.

## 5 Conclusions

Our experiment provides valuable insights into how EMDR stimulation might mitigate the effects of intense stress. We demonstrated that dendritic debranching/remodeling in hippocampus represents a core target for the benefits of EMDR. These findings may offer neurobiological insights into the therapeutic advantages of any procedure that incorporates EMDR stimulation.

## Data availability statement

The raw data supporting the conclusions of this article will be made available by the authors, without undue reservation.

## Ethics statement

The animal study was approved by Health Sciences Ethics, Biosafety, and Scientific Board, at the University of Guadalajara. The study was conducted in accordance with the local legislation and institutional requirements.

## Author contributions

YR-D: Writing – review & editing, Writing – original draft, Project administration, Methodology, Funding acquisition, Formal analysis, Conceptualization. DM-F: Writing – review & editing, Software, Methodology. SL: Writing – review & editing, Supervision, Resources. AM-A: Writing – review & editing, Supervision, Conceptualization. DR-R: Writing – review & editing, Supervision, Conceptualization. FJ-H: Writing – review & editing, Validation, Resources, Investigation, Formal analysis. DF-Q: Writing – review & editing, Writing – original draft, Software, Project administration, Methodology, Investigation, Funding acquisition, Formal analysis, Conceptualization.
